# Social trust and depression among young adults in Seoul, South Korea: The moderating effect of traditional gender role attitudes

**DOI:** 10.1371/journal.pone.0355298

**Published:** 2026-08-28

**Authors:** Sangmi Lee

**Affiliations:** Department of Nursing, Dongyang University, Yeongju-si, Gyeongsangbuk-do, Republic of Korea; Regional Health Care and Social Agency of Lodi, ITALY

## Abstract

This study examined the association between social trust and depression among young adults in Seoul, South Korea, and investigated the moderating role of traditional gender role attitudes. Data were obtained from 592 young adults aged 19–34 years who participated in the 2022 Seoul Mental Health Survey. A cross-sectional secondary analysis was conducted using SPSS 28.0 and Hayes’ PROCESS Macro (Model 1), controlling for gender, educational attainment, economic activity, and living arrangement. Social trust was negatively associated with depression (B = −0.210, p < .001), whereas traditional gender role attitudes were positively associated with depression (B = 0.111, p = .001). The interaction between social trust and traditional gender role attitudes was significant (B = 0.189, p < .001), explaining an additional 2.5% of the variance in depression (ΔR² = .025). The negative association between social trust and depression was significant when traditional gender role attitudes were low (mean − 1 SD) or average, but not when they were high (mean + 1 SD). Johnson–Neyman analysis indicated that the association remained significant when traditional gender role attitude scores were below 2.92. These findings suggest that the association between social trust and depression varies according to traditional gender role attitudes. Social trust may be more strongly associated with lower depressive symptoms among young adults with lower endorsement of traditional gender role attitudes.

## Introduction

### Background

Depression is one of the most common mental health problems worldwide and a leading contributor to disability and reduced quality of life across populations [1]. According to the World Health Organization, depression affects hundreds of millions of people globally and represents a major public health challenge due to its substantial social, economic, and health-related burden [1]. Although depression is prevalent across all regions and age groups, its distribution and associated risk factors vary according to sociocultural and environmental contexts. In Korea, the prevalence of depressive symptoms among adults remains high, with 11.6% reporting such experiences in 2023 [2]. In the Korean context, young adulthood is commonly defined as ages 19–34 years, reflecting the prolonged transition to stable employment, marriage, and family formation in contemporary society [3,4]. Young adulthood is a critical transitional stage characterized by major life challenges, including educational attainment, employment and workplace adaptation, marriage, and family formation. These transitions are often accompanied by increased psychosocial stress and adjustment demands [5,6]. Reflecting these characteristics, the prevalence of depressive symptoms is highest among young adults, with 16.3% in the 19–29 age group and 11.6% in the 30–39 age group, indicating the highest levels of depression across adulthood [2].

Mental health is shaped not only by individual psychological traits but also by environmental factors [7–9]. Among these, social trust has been highlighted as an important form of social capital [7,8]. Social trust refers to a general belief in the reliability of others and society at large. Individuals with high levels of social trust are more likely to access social support, feel a sense of community belonging, and maintain psychological stability. These characteristics have been identified as protective factors against depression in several studies [10]. Particularly during young adulthood, when individuals are engaged in identity formation, role exploration, and autonomy development, social trust is reported to contribute substantially to mental health [11–13]. Social trust is a core component of social capital theory and facilitates cooperation, reciprocity, and access to supportive social networks. Through these mechanisms, social trust may buffer psychological stress and promote emotional well-being [7,8,10].

Social values also represent critical environmental factors for mental health. Although social trust can provide access to social resources and psychological support, the extent to which individuals perceive, utilize, and benefit from these resources may depend on their personal values and social beliefs. Among these, traditional gender role attitudes may shape how individuals interpret social relationships, seek support, and respond to psychosocial stressors [14–16]. Traditional gender role attitudes may hinder adaptation to social change and intensify role conflict or strain, which may be associated with higher levels of depression, anxiety, and stress [17–20]. Traditional gender role attitudes are socially and culturally acquired and become internalized over time, exerting long-term influence on behaviors and attitudes. Such rigid gender norms may negatively affect mental health and overall quality of life [21].

Although social trust generally functions as a protective factor against depression, its effect may vary depending on individual values, particularly traditional gender role attitudes. Previous studies have shown that gender role stereotypes can moderate the relationship between stress and depression, such that stronger traditional gender role stereotypes intensified the impact of stress on depression [22]. Therefore, gender role attitudes should be considered not merely as personal traits but as key cultural and social factors shaping the relationship between social trust and mental health. Individuals with more traditional gender role attitudes may be more likely to endorse rigid expectations regarding appropriate roles, emotional expression, and interpersonal behavior [23]. Such attitudes may discourage emotional disclosure and help-seeking behaviors and limit the extent to which individuals utilize or benefit from social resources embedded in trusting social relationships [24,25].

Traditional gender role attitudes were conceptualized as a contextual attitudinal framework that may influence how individuals perceive and benefit from social resources. Whereas social trust reflects a social resource that facilitates social integration and access to support [7,8,10], gender role attitudes reflect socially acquired beliefs and expectations regarding appropriate roles and behaviors for men and women [17–21]. Previous studies have suggested that gender-related attitudes and norms can shape individuals’ psychological responses to social environments and resources [22,26,27]. Accordingly, gender role attitudes may influence how individuals perceive and utilize available social resources, including trust-based social relationships. Therefore, rather than serving as an intermediary mechanism through which social trust affects depression, gender role attitudes were expected to alter the strength of this association. For this reason, a moderation model was considered more theoretically appropriate than a mediation model.

Furthermore, Korean society is currently undergoing a transitional period in which remnants of traditional gender role norms coexist with the expansion of gender equality values [28,29]. Young adults, in particular, are in the process of reconstructing their gender role values in response to these societal changes [30], which may directly affect how they mobilize social trust as a psychological resource. If traditional gender role attitudes weaken the negative association between social trust and depression, future intervention research may need to consider both trust-related resources and gender role attitudes. These findings may suggest the potential value of considering both social trust and gender role attitudes when developing future mental health promotion strategies for young adults.

However, most previous studies on traditional gender role attitudes and depression have examined general adult populations, including young adults, without focusing exclusively on this age group [31–33]. Although several studies have explored the moderating role of gender-related attitudes in mental health outcomes, limited attention has been paid to whether gender role attitudes shape the association between social trust and depression. In particular, little is known about this relationship among young adults in the Korean sociocultural context, where traditional gender norms continue to coexist with rapid social change.

Therefore, this study aims to investigate the relationship between social trust and depression among young adults and to test the moderating effect of traditional gender role attitudes. By doing so, it seeks to move beyond simple gender comparisons, provide a more nuanced understanding of psychosocial factors influencing young adults’ mental health, and contribute to the development of effective intervention strategies for the prevention of depression and the promotion of mental health in this population.

### Objectives and hypotheses

This study examined whether traditional gender role attitudes moderate the association between social trust and depression among young adults in Seoul, South Korea. Based on the theoretical framework and previous literature, the following hypotheses were proposed:

Hypothesis 1 (H1): Higher levels of social trust will be associated with lower levels of depression.

Hypothesis 2 (H2): Stronger traditional gender role attitudes will be associated with higher levels of depression.

Hypothesis 3 (H3): Traditional gender role attitudes will moderate the relationship between social trust and depression, such that the protective effect of social trust against depression will vary across levels of traditional gender role attitudes.

## Literature review

### The relationship between social trust and depression

Social trust, as a core factor in forming social bonds through mutual trust, cooperation, and a sense of belonging among members of society, serves as an essential psychosocial resource that significantly influences psychological well-being and mental health [10]. Higher levels of social trust have been associated with stronger social relationships, greater emotional stability, and lower depressive symptoms. Several studies have shown that social trust is negatively associated with depression among young adults, with the effect being stronger in women [11]. In addition, the perception of social conflict has been found to increase depression, whereas social trust mediated this relationship, alleviating depressive symptoms even in groups with high conflict perception when their trust levels were high [13]. Similarly, in the relationship between anomie and depression among young adults, social trust demonstrated a partial mediating effect, with higher levels of trust linked to lower depression [34].

The protective effect of social trust also appears to interact with young adults’ socioeconomic perceptions. For instance, the impact of subjective class perception on depression varied by levels of social trust, with stronger negative effects observed in groups with lower trust [35]. Moreover, research on socially withdrawn youth indicated that higher social trust was significantly associated with reduced depression [12]. These findings suggest that social trust plays a buffering role in the vulnerable psychological states of young adults. International evidence also supports the role of social trust as a protective factor against depression. A Swedish cohort study found that generalized trust during adolescence was negatively associated with depressive and anxiety symptoms in early adulthood (20–21 years old) [36]. In Montreal, Canada, generalized trust and neighborhood trust were linked to a lower likelihood of depressive symptoms [7]. A large-scale panel study in Indonesia revealed that higher social trust reduced the risk of depressive symptoms, while lower neighborhood trust increased the risk of severe depression [8]. Similarly, an analysis from the United States reported that lower institutional trust was associated with higher depressive symptoms [37].

Further, some studies suggest neurobiological mechanisms underlying the association between social trust and depression. For example, individuals with low levels of social trust showed reduced gray matter volume in brain regions such as the prefrontal cortex, which has been linked to vulnerability to depression [38]. Recent longitudinal evidence also supports the protective role of social trust across the lifespan. Wang et al. [39] found that older adults with consistently high social trust reported significantly lower levels of depressive symptoms over time, with the protective effect being particularly pronounced among urban residents. Among migrant adolescents in China, interpersonal trust, as a form of social capital, was negatively associated with depression, indicating that the protective role of social trust transcends cultural and national boundaries [40]. In summary, social trust functions as a key psychosocial resource in alleviating depression among young adults, with its protective effect confirmed across various sociocultural contexts and socioeconomic conditions. Specifically, social trust has been shown to buffer against diverse psychosocial risk factors among youth, including conflict perception, anomie, class perception, and social withdrawal, as consistently reported in numerous previous studies.

### The relationship between traditional gender role attitudes and depression

Traditional gender role attitudes, which prescribe rigid roles and behavioral norms for men and women, often encompass gender-discriminatory elements. These attitudes influence self-identity formation, interpersonal relationship styles, and the utilization of social resources, thereby holding significant implications for mental health [41]. Prior research has consistently shown that stronger traditional gender role attitudes are associated with greater psychological burdens, manifesting differently by gender, and ultimately contributing to poorer mental health outcomes.

For women, traditional role norms such as sacrifice, obedience, and caregiving responsibilities amplify social pressure to conform to expectations, which can cause psychological distress. A study of women in Kosovo reported that higher levels of feminine role stress were associated with increased psychological distress [18]. Similarly, a Korean study found that traditional gender role attitudes among women aged 20–50 were positively correlated with depression and remained significant after controlling for age, education, religion, and employment status [32].

For men, normative expectations such as toughness and emotional suppression may restrict emotional expression and the use of social networks, thereby exacerbating mental health difficulties. A study on Korean adults aged 20–40 [33] found that stronger traditional gender role attitudes were associated with higher levels of depression, with particularly pronounced effects among men in their 20s and 30s. Gender role conflict has also been shown to negatively impact men’s mental health: for instance, higher levels of role conflict were linked to greater depression among men in their 20s [20], with the impact varying depending on levels of social support [42]. Recent evidence further highlights the complex relationship between gender role attitudes and men’s mental health. Yang [31] reported that Korean men who exhibited lower conformity to traditional gender role norms experienced the highest levels of depression, suggesting that deviations from socially prescribed masculine expectations may also generate psychological distress. In addition, a recent review concluded that traditional masculine norms often discourage help-seeking behaviors through emotional suppression and excessive self-reliance, thereby increasing vulnerability to mental health problems [25].

The association between traditional gender role attitudes and depression has been documented across diverse cultural contexts and age groups. For example, a study of adolescents in Shanghai [17] found that gender-stereotypical traits and perceptions of sexual double standards were related to depression, while research in the Netherlands demonstrated that higher masculinity norm scores among adults were associated with increased depression and stress [19]. Similarly, traditional masculine norm adherence was linked to greater depression among Korean [31] and Israeli men [43], with patriarchal family environments exacerbating depression through gender role conflict [44]. Among married women living in poverty, both traditional gender role attitudes and depression levels were higher compared to their non-poor counterparts [45]. Studies in Western contexts have also found that perceptions of gender inequality heightened the risk of depression and anxiety [14]. Similarly, recent findings from China showed that egalitarian gender role attitudes were associated with better psychological well-being, with the beneficial effects being more pronounced among men than women [46]. These findings suggest that traditional gender role attitudes may adversely affect mental health not only by reinforcing restrictive social expectations but also by limiting psychological flexibility and adaptive coping strategies. In addition, research on Australian adolescents revealed that egalitarian gender role attitudes were negatively associated with behavioral problems and positively associated with prosocial behavior, suggesting that gender role attitudes are related to mental health not only in adults but also in adolescents [15]. Taken together, these findings suggest that traditional gender role attitudes have been associated with depressive symptoms across diverse gender, age, and cultural groups.

### The moderating role of traditional gender role attitudes

Traditional gender role attitudes are not only direct risk factors for depression but may also function as moderators in the relationship between other psychosocial factors and depression. For example, Jwa [22] found that traditional feminine gender role stereotypes intensified the association between academic stress and depression among adolescents, while Son & Yoo [26] reported that traditional gender role attitudes moderated the relationship between future orientation and interpersonal need frustration related to suicide among female college students. A study exploring the relationships among gender roles, maternal attitudes, and mental health in Mexican women [16] found that higher internalization of traditional gender role attitudes was associated with more rigid maternal attitudes, which in turn increased anxiety and emotional stress. Conversely, gender role flexibility acted as a protective factor, fostering healthier maternal attitudes and promoting positive mental health.

From the perspective of social capital theory, social trust enhances access to resources and mutual support through interpersonal networks, thereby mitigating depression [7,8,10]. Traditional gender role attitudes may shape how individuals perceive and utilize social relationships and support resources [23,24]. Individuals with stronger traditional gender role attitudes may be less likely to seek emotional support or engage with broader social networks, whereas those with more egalitarian attitudes may be more open to diverse interpersonal relationships and sources of support [23,25,42]. Consequently, the mental health benefits derived from social trust may vary according to individuals’ gender role attitudes. Previous studies have shown that gender role attitudes can moderate the effects of psychosocial and environmental factors on mental health [16,22,26]. Such findings support the possibility that traditional gender role attitudes may influence the extent to which psychosocial resources, including social trust, protect against depression.

**Fig 1:**
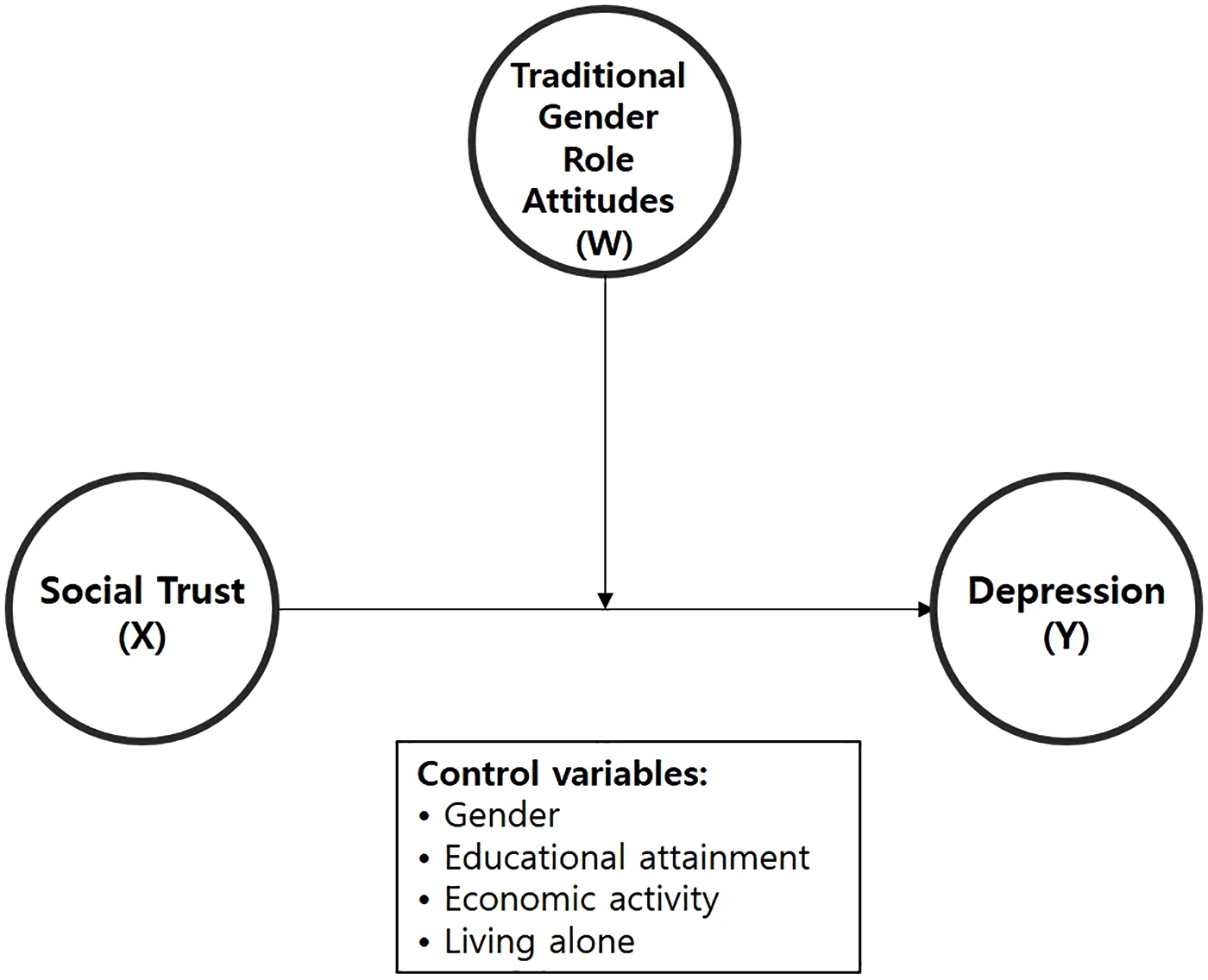
Conceptual model of the moderating effect of traditional gender role attitudes on the association between social trust and depression.

Therefore, traditional gender role attitudes may serve as a moderating factor in the relationship between social trust and depression. Examining this moderating effect among young adults provides important foundational evidence for designing tailored mental health interventions. Based on a synthesis of prior research and literature, the research model was developed as shown in Fig 1.

## Methods

### Research design and ethical considerations

This study was a secondary analysis of publicly available, de-identified data from the 2022 Mental Health Survey of Seoul Citizens. The Institutional Review Board of Dongyang University determined that this secondary analysis was exempt from ethical review (IRB No. 202509−001). Because the dataset contained no personally identifiable information and was publicly available for research use through the Korean Social Science Data Archive (KOSSDA), informed consent was not required.

### Data and participants

This study used raw data from the 2022 Seoul Mental Health Survey conducted by the Seoul Foundation of Women & Family [47]. The survey was administered from April 13–19, 2022, targeting adults aged 19 years and older residing in Seoul. Based on the Ministry of the Interior and Safety’s resident registration population statistics as of March 2022, 2,000 participants were quota-sampled to reflect population proportions, and data were collected through an online survey. The structured questionnaire included items on social perceptions, interpersonal and family relationships, health status, and experiences with mental health services to assess the mental health status and policy needs of Seoul citizens.

The inclusion criteria for the present analysis were: (1) participation in the 2022 Seoul Mental Health Survey, (2) age between 19 and 34 years, and (3) valid responses on social trust, traditional gender role attitudes, depression, and covariates. Participants outside this age range were excluded. Because no missing values were identified for the study variables, no additional cases were excluded due to incomplete responses. Consequently, 592 participants aged 19–34 years were included in the final analysis. In this study, young adulthood was defined as ages 19–34 years in accordance with the Korean Youth Basic Act, which defines youth as individuals aged 19–34 years [3]. This age range has also been adopted in recent Korean studies examining the mental health and psychosocial experiences of young adults [4].

The dataset used in this study is publicly available through the KOSSDA under the title *Mental Health Survey of Seoul Citizens, 2022* (https://doi.org/10.22687/KOSSDA-A1-2022-0103-V1). The data were obtained through KOSSDA after researcher registration, agreement to the archive’s data-use regulations, and submission of the research purpose. The public-use dataset did not provide sampling weights; therefore, all analyses were conducted using unweighted data. A sensitivity power analysis using G*Power 3.1 indicated that, with α = .05, power = .80, a sample size of 592, seven predictors, and one tested interaction term, the study had sufficient statistical power to detect an effect size of f² = .013 or larger in the R² increase test.

## Measures

The measures were drawn from the 2022 Seoul Mental Health Survey [47] and included the following variables: social trust (independent variable), depression (dependent variable), and traditional gender role attitudes (moderator). The full wording of the social trust and traditional gender role attitude items is presented in S1 Table and S2 Table.

### Social trust

Social trust was measured across nine domains: family, friends or neighbors, coworkers, strangers, the central government, local government, corporations, NGOs, and the media. Each item was rated on a five-point Likert scale (1 = “do not trust at all,” 5 = “trust very much”), with higher mean scores indicating higher levels of social trust. The reliability of this scale in the current study was Cronbach’s α = .824.

### Traditional gender role attitudes

Traditional gender role attitudes were assessed using six items that measured perceptions of gender-specific roles in livelihood, emotional expression, childcare, housework, social participation, and leadership. Items included statements such as “The primary responsibility for family livelihood lies with men,” reflecting traditional gender role expectations. Each item was rated on a five-point Likert scale (1 = “strongly disagree,” 5 = “strongly agree”), with higher mean scores indicating stronger adherence to traditional gender role attitudes. The reliability of this scale in the current study was Cronbach’s α = .888. Construct validity was examined using exploratory factor analysis with principal axis factoring. The Kaiser–Meyer–Olkin measure of sampling adequacy was .852, and Bartlett’s test of sphericity was significant (χ² = 2019.552, df = 15, p < .001). One factor with an eigenvalue of 3.454 was extracted, accounting for 57.57% of the total variance. Factor loadings ranged from .652 to .853, supporting the unidimensional structure of the scale.

### Depression

Depression was assessed using the Patient Health Questionnaire-9 (PHQ-9), which consists of nine items. Items included “Feeling down, depressed, or hopeless,” “Little interest or pleasure in doing things,” and “Thoughts that you would be better off dead or of hurting yourself.” Respondents rated the frequency of symptoms over the past two weeks on a four-point scale (0 = “not at all,” 1 = “several days,” 2 = “more than half the days,” 3 = “nearly every day”). Higher scores indicated greater depressive symptoms. The reliability of this scale in the current study was Cronbach’s α = .916.

### Control variables

Based on previous studies, four control variables were included: gender, educational attainment, economic activity in the past week, and living arrangement (living alone). Prior research has shown that depression tends to be higher among women [19,33], those with lower educational attainment [11,13], those with lower socioeconomic status [35,45], and those living without family members [20]. Gender was coded as female = 0, male = 1. Educational attainment was recoded into three levels (≤ high school = 1, 2–4 year college = 2, ≥ graduate school = 3) and treated as an ordinal variable. Economic activity was coded as yes = 1, no = 0, and living alone as yes = 1, no = 0.

### Data analysis

Data analyses were performed using SPSS 28.0 and PROCESS Macro version 4.2 developed by Hayes [48]. Prior to analysis, missing data were examined for all study variables. No missing values were identified for the variables included in the analyses; therefore, all 592 eligible participants were retained in the final analytic sample. Normality assumptions were assessed by examining skewness and kurtosis values. The absolute values of skewness ranged from 0.11 to 1.17, and the absolute values of kurtosis ranged from 0.44 to 0.87, indicating acceptable normality [49].

Descriptive statistics were used to examine participants’ sociodemographic characteristics and the distribution of study variables. Pearson correlation coefficients were calculated to examine associations among variables. Prior to the moderation analysis, multicollinearity was assessed using tolerance and variance inflation factor (VIF) values, and the independence of residuals was examined using the Durbin–Watson statistic. Tolerance values ranged from 0.875 to 0.993, VIF values ranged from 1.007 to 1.143, and the Durbin–Watson statistic was 2.154, indicating no concerns regarding multicollinearity or autocorrelation. To test the moderating effect of traditional gender role attitudes on the relationship between social trust and depression, PROCESS Macro Model 1 was applied. Social trust and traditional gender role attitudes were mean-centered before creating the interaction term. In addition, standardized residual plots and normal P–P plots were examined to assess the assumptions of linearity, homoscedasticity, and normality of residuals. Visual inspection indicated that these assumptions were reasonably satisfied, with no substantial deviations observed. To assess common method bias, Harman’s single-factor test was conducted using all measurement items. The first unrotated factor explained 23.93% of the total variance, which was below the 50% criterion, suggesting that common method bias was not a serious concern [50]. To facilitate interpretation of the relative strength of the key predictors, additional analyses using standardized variables were conducted and standardized regression coefficients (β) were examined.

## Results

### Characteristics of the participants

The mean age of the participants was 27.85 years (SD = 3.55), with ages ranging from 19 to 34 years. Among the 592 participants, 315 were women (53.2%) and 277 were men (46.8%). Regarding educational attainment, the majority had completed a 2–4 year college education (n = 446, 75.3%), followed by high school or less (n = 113, 19.1%) and graduate school or higher (n = 33, 5.6%). A total of 430 participants (72.6%) reported engaging in income-generating activities during the past week, and 202 (34.1%) lived alone. The mean scores were 2.83 (SD = 0.59) for social trust, 2.33 (SD = 0.92) for traditional gender role attitudes, and 0.72 (SD = 0.69) for depression (Table 1).

**Table 1:** Sociodemographic and psychological characteristics of the participants (N = 592).

Characteristics		Category	n (%)	Mean ± SD	Range
**Sociodemographic characteristics**	**Age (years)**	–	–	27.85 ± 3.55	19.00-34.00
	**Gender**	Women	315 (53.2)	–	–
		Men	277 (46.8)	–	–
	**Educational attainment**	≤ High school	113 (19.1)	–	–
		2-4 year college	446 (75.3)	–	–
		≥ Graduate school	33 (5.6)	–	–
	**Economic activity**	Yes	430 (72.6)	–	–
		No	162 (27.4)	–	–
	**Living arrangement**	Living alone	202 (34.1)	–	–
		Not living alone	390 (65.9)	–	–
**Psychological variables**	**Social trust**	–	–	2.83 ± 0.59	1.00-5.00
	**Traditional gender role attitudes**	–	–	2.33 ± 0.92	1.00-5.00
	**Depression**	–	–	0.72 ± 0.69	0.00-3.00

Gender differences are presented in S3 Table. Men reported significantly higher levels of social trust (t = −2.37, p = .018) and traditional gender role attitudes (t = −6.90, p < .001) than women. In contrast, women reported significantly higher levels of depression than men (t = 3.32, p = .001).

### Correlations among key variables

The correlations among the key variables are presented in Table 2. Social trust was positively correlated with traditional gender role attitudes (r = .244, p < .001) and negatively correlated with depression (r = −.123, p = .003). Traditional gender role attitudes were also positively correlated with depression (r = .095, p = .021). Among the control variables, gender showed significant positive correlations with both social trust (r = .097, p = .018) and traditional gender role attitudes (r = .273, p < .001), and a significant negative correlation with depression (r = −.136, p = .001). This indicates that men reported higher levels of social trust and traditional gender role attitudes but lower levels of depression compared to women. Educational attainment was negatively correlated with traditional gender role attitudes (r = −.083, p = .044) and depression (r = −.090, p = .029), suggesting that individuals with higher educational attainment tended to report lower endorsement of traditional gender role attitudes and lower levels of depressive symptoms. Educational attainment was also negatively correlated with gender (r = −.202, p < .001), indicating that higher educational attainment was more common among women. In addition, economic activity was positively correlated with educational attainment (r = .286, p < .001), indicating that participants engaged in income-generating activities tended to have higher educational attainment. No significant correlations were observed for living arrangement.

**Table 2:** Correlations among study variables (N = 592).

r (p)	Social trust	Traditional gender role attitudes	Depression	Gender	Educational attainment	Economic activity
**Social trust**	1					
**Traditional gender role attitudes**	.244 (<.001)	1				
**Depression**	−.123 (.003)	.095 (.021)	1			
**Gender**	.097 (.018)	.273 (<.001)	−.136 (.001)	1		
**Educational attainment**	.026 (.532)	−.083 (.044)	−.090 (.029)	−.202 (<.001)	1	
**Economic activity**	−.014 (.725)	−.065 (.115)	−.063 (.124)	−.070 (.089)	.286 (<.001)	1
**Living alone**	−.023 (.581)	−.029 (.480)	.003 (.950)	−.011 (.793)	.039 (.337)	−.074 (.071)

Values are Pearson correlation coefficients (r), with p-values shown in parentheses.

^a^Gender was coded as female = 0 and male = 1.

^b^Educational attainment was coded as 1 = ≤ high school, 2 = 2–4 year college, and 3 = ≥ graduate school.

^c^Economic activity was coded as yes = 1 and no = 0, referring to income-generating activity during the past week.

^d^Living alone was coded as yes = 1 and no = 0.

### Hypothesis testing

To examine whether traditional gender role attitudes moderated the relationship between social trust and depression, Hayes’ [48] moderation model (Model 1) was applied (Table 3). The overall model was statistically significant (F = 8.86, p < .001), explaining 9.6% of the variance in depression (R² = .096). The interaction term accounted for an additional 2.5% of the variance in depression (ΔR² = .025), corresponding to a small effect size (Cohen’s f² = .028) according to Cohen’s [51] criteria.

**Table 3:** The moderating role of traditional gender role attitudes in the association between social trust and depression.

Predictor	B	SE	t	p	LLCI	ULCI
**Social trust**	−.210	.049	−4.327	<.001	−.305	−.115
**Traditional gender role attitudes**	.111	.032	3.483	.001	.048	.173
**Social trust** * **Traditional gender role attitudes**	.189	.047	3.993	<.001	.096	.282
**Gender**	−.241	.058	−4.160	<.001	−.355	−.127
**Educational attainment**	−.140	.061	−2.313	.021	−.259	−.021
**Economic activity**	−.053	.064	−0.832	.406	−.178	.072
**Living alone**	.003	.058	0.060	.952	−.110	.117
Model statistics: F = 8.86, p < .001, R^2^ = .096, △R^2^ = .025, Cohen’s f² = .028.						

B = unstandardized regression coefficient; SE = standard error; LLCI = lower limit of the 95% confidence interval; ULCI = upper limit of the 95% confidence interval.

^a^Gender was coded as female = 0 and male = 1.

^b^Educational attainment was coded as 1 = ≤ high school, 2 = 2–4 year college, and 3 = ≥ graduate school.

^c^Economic activity was coded as yes = 1 and no = 0.

^d^Living alone was coded as yes = 1 and no = 0.

The results showed that social trust had a significant negative association with depression (B = –.210, p < .001), supporting Hypothesis 1 that higher levels of social trust would be associated with lower levels of depression. Traditional gender role attitudes had a significant positive association with depression (B = .111, p = .001), supporting Hypothesis 2 that stronger endorsement of traditional gender role attitudes would be associated with higher levels of depressive symptoms. After mean-centering the independent variable (social trust) and moderator (traditional gender role attitudes) and including the interaction term, the interaction was found to be statistically significant (B = .189, p < .001), supporting Hypothesis 3 and confirming the moderating effect of traditional gender role attitudes. Additional analyses using standardized variables showed that the corresponding standardized coefficients were β = –.193 for social trust, β = .159 for traditional gender role attitudes, and β = .153 for the interaction term.

Among the control variables, economic activity and living arrangement were not significantly associated with depression. However, gender (B = –.241, p < .001) and educational attainment (B = –.140, p = .021) were significantly negatively associated with depression, indicating that men and individuals with higher educational attainment reported lower levels of depression.

**Fig 2:**
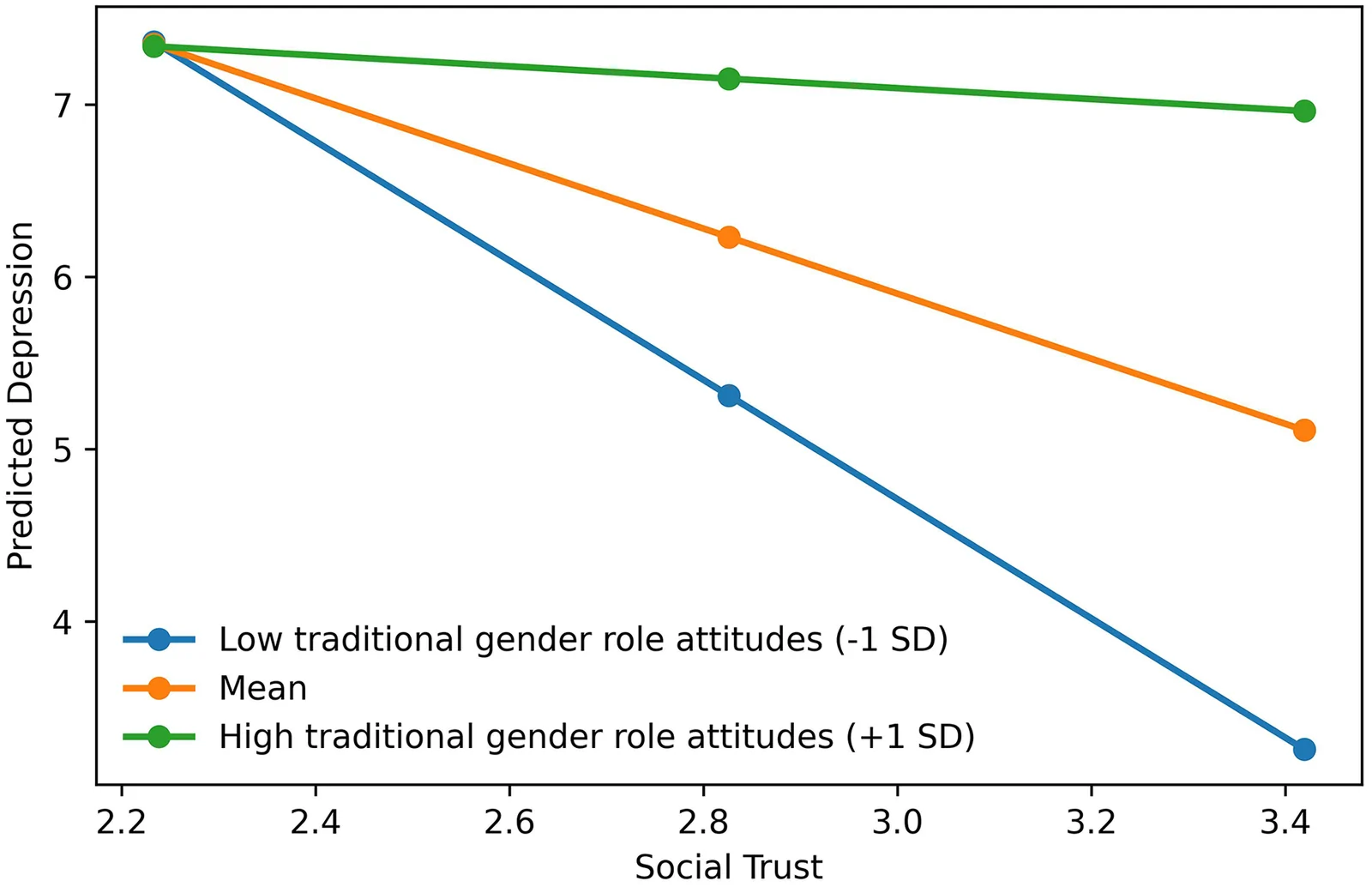
Interaction effect of social trust and traditional gender role attitudes on depression. Lines represent estimated associations between social trust and depression at low (mean − 1 SD), average, and high (mean + 1 SD) levels of traditional gender role attitudes.

The conditional effects of social trust on depression at different levels of traditional gender role attitudes are presented in Table 4 and Fig 2 [52]. When the level of traditional gender role attitudes was low (mean − 1 SD), social trust had a significant negative effect on depression (B = −.385, p < .001), indicating that higher levels of social trust were associated with significantly lower levels of depression. At the mean level of traditional gender role attitudes, social trust remained negatively associated with depression (B = −.210, p < .001), although the magnitude of the coefficient was smaller than that observed at low levels of traditional gender role attitudes. In contrast, at the high level of traditional gender role attitudes (mean + 1 SD), the relationship between social trust and depression was not statistically significant (B = −.035, p = .543). As illustrated in Fig 2, the negative slope between social trust and depression was steepest at low levels of traditional gender role attitudes and became progressively flatter as traditional gender role attitudes increased.

**Table 4:** Conditional effects of social trust on depression across levels of traditional gender role attitudes.

Levels	Effect	SE	t	p	LLCI	ULCI
**Mean –1 SD**	−.385	.072	−5.338	<.001	−.526	−.243
**Mean**	−.210	.049	−4.327	<.001	−.305	−.115
**Mean +1 SD**	−.035	.058	−.609	.543	−.149	.078

Conditional effects of social trust on depression at low (mean − 1 SD), moderate (mean), and high (mean + 1 SD) levels of traditional gender role attitudes. LLCI = lower limit of the 95% confidence interval; ULCI = upper limit of the 95% confidence interval.

**Fig 3:**
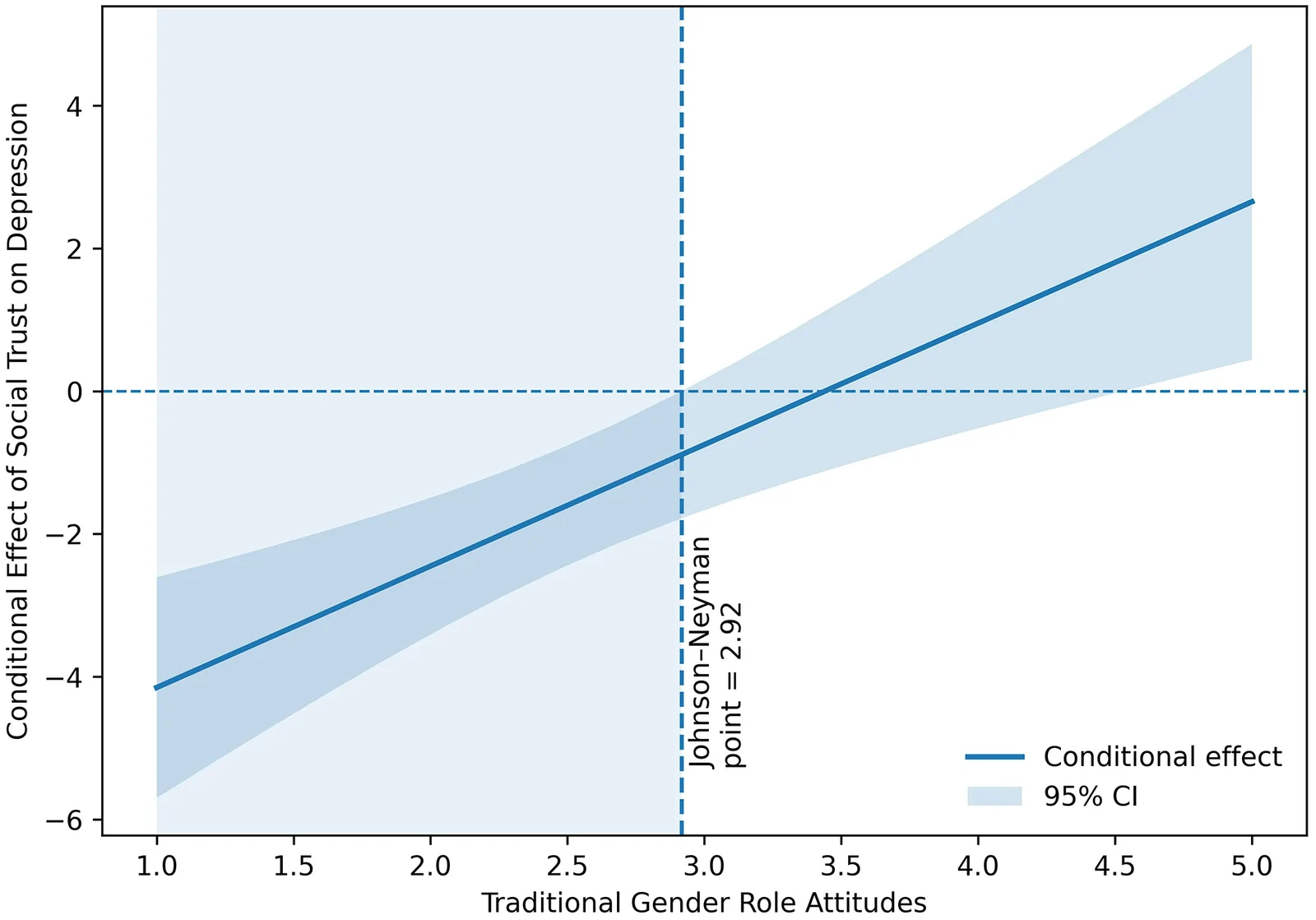
Johnson–Neyman plot of the conditional effect of social trust on depression across levels of traditional gender role attitudes. The shaded area indicates the region in which the conditional effect was statistically significant.

As shown in Fig 3, Johnson–Neyman analysis indicated that the association between social trust and depression remained statistically significant when traditional gender role attitude scores were below 2.92. This region included approximately 69.3% of the sample. Above this value, the association was no longer statistically significant.

## Discussion

The present study examined the relationship between social trust and depression among young adults in Seoul, South Korea and investigated the moderating role of traditional gender role attitudes. The results showed that higher levels of social trust were associated with lower levels of depression, whereas stronger endorsement of traditional gender role attitudes was associated with higher levels of depressive symptoms. Furthermore, the negative association between social trust and depression was weaker among individuals with higher levels of traditional gender role attitudes.

First, social trust was significantly associated with lower levels of depression among young adults (B = –.210, p < .001), supporting Hypothesis 1. This finding is consistent with previous studies reporting that social trust is associated with lower levels of depressive symptoms and better psychological well-being among young people [11,13,34,36]. These findings support the view that social trust is an important social resource linked to depressive symptoms and emotional well-being during young adulthood.

Second, traditional gender role attitudes were positively associated with depression (B = .111, p = .001), supporting Hypothesis 2 and suggesting that stronger endorsement of rigid gender role norms may be related to greater psychological distress. This finding is consistent with previous studies conducted among older women [27], general adults [31–33], and adolescents [17], which reported positive associations between traditional gender role attitudes and depressive symptoms. The present study extends these findings to young adults and suggests that traditional gender role attitudes may represent a psychosocial factor associated with depression in this population. However, the mechanisms underlying this relationship were not directly examined and should be investigated in future research.

Third, traditional gender role attitudes significantly moderated the relationship between social trust and depression (B = .189, p < .001), supporting Hypothesis 3. The negative association between social trust and depression was stronger among individuals with lower levels of traditional gender role attitudes and weaker among those with higher levels of endorsement. This finding is consistent with previous studies suggesting that rigid gender role attitudes may be associated with greater psychological vulnerability [22,26], whereas Delgado-Herrera et al. [16] reported that gender role flexibility was associated with more favorable psychological adjustment. The findings further suggest that the mental health relevance of social trust may vary according to individuals’ gender role attitudes.

The associations observed for gender and educational attainment also warrant comment. Female participants reported higher levels of depression than male participants, consistent with previous national and international findings [1,17,19,33]. Although the reasons for this gender difference were not examined in the present study, previous studies have suggested that gender role expectations, gender role conflict, and gender-related social experiences may contribute to disparities in mental health outcomes [14,31,33,44]. In addition, higher educational attainment was associated with lower levels of depressive symptoms, supporting previous research indicating an inverse relationship between education and depression [11,13]. Higher educational attainment may be associated with greater access to socioeconomic resources and opportunities that support psychological well-being, although these mechanisms were not directly examined in the present study. Taken together, these findings suggest that sociodemographic factors may play an important role in young adults’ mental health and warrant further investigation.

By examining both social trust and traditional gender role attitudes, this study contributes to a broader understanding of psychosocial factors associated with depression among young adults. Previous studies have examined the relationship between social trust and depression, including gender differences and related psychosocial mechanisms [11,13,34]. However, the present study extends the literature by examining traditional gender role attitudes as a psychosocial moderator. The findings suggest that cultural and ideological value systems may influence how social resources are associated with mental health outcomes, highlighting the need for further research using longitudinal and intervention-based designs.

These findings have several practical implications. First, social trust represents an important psychosocial resource associated with lower levels of depressive symptoms among young adults. Accordingly, interventions aimed at strengthening social trust may be considered in future mental health promotion efforts. Examples include peer-support groups, university-based mentoring programs, volunteer activities, and community engagement projects. Broader efforts to foster transparent, inclusive, and supportive social environments may also contribute to young adults’ mental health. However, future intervention studies are needed to evaluate whether enhancing social trust leads to measurable improvements in mental health outcomes.

Second, the results indicate that traditional gender role attitudes may influence the extent to which young adults benefit from social trust. Accordingly, programs that encourage young adults to critically examine traditional gender role norms and promote more flexible and egalitarian gender role attitudes, such as gender equality workshops, psychoeducational programs, and campus-based mental health initiatives addressing gender-related stressors, may be considered in future mental health promotion initiatives. Nevertheless, because the present study did not directly examine intervention effects or underlying mechanisms, such recommendations should be interpreted cautiously and require empirical evaluation. Third, the significant associations of gender and educational attainment with depression highlight the potential importance of sociodemographic inequalities in young adults’ mental health. Future policies and programs may benefit from taking into account the social and educational contexts that shape young adults’ experiences of psychological distress and access to mental health resources.

Despite its contributions, this study has several limitations. First, the cross-sectional design precludes causal inferences regarding the relationships among social trust, traditional gender role attitudes, and depression. Reverse causality cannot be ruled out, as individuals experiencing higher levels of depressive symptoms may perceive lower levels of social trust or endorse more rigid gender role attitudes. Future longitudinal studies are needed to clarify the directionality of these relationships. Second, all variables were measured using self-report questionnaires, which may be subject to response bias and common method bias. Future studies should incorporate multiple data sources and longitudinal assessments to reduce potential measurement bias. Third, the sample was limited to young adults residing in Seoul. Because Seoul represents a highly urbanized social and cultural context, the findings may not be generalizable to young adults living in other regions of South Korea. Fourth, young adulthood was defined as ages 19–34 years based on the Korean Youth Basic Act and the Korean sociocultural context. Therefore, caution should be exercised when comparing the findings with studies that use narrower age ranges for young adulthood. Finally, depression was assessed using the PHQ-9, a widely used screening instrument for depressive symptoms rather than a clinical diagnostic tool. Future studies should examine whether similar findings are observed using clinical diagnoses or longitudinal measures of depression.

## Supporting information

S1 Table Social trust items. (DOCX)

S2 Table Traditional gender role attitude items. (DOCX)

S3 Table Gender differences in key study variables. (DOCX)
