# Supplementary material for: Social trust and depression among young adults in Seoul, South Korea: The moderating effect of traditional gender role attitudes
Source: PLoS One. 2026 Aug 28;21(8):e0355298. doi: 10.1371/journal.pone.0355298 (PMC13524275; doi:10.1371/journal.pone.0355298)
Supplement: Supplementary file 1 [file pone.0355298.s001.docx]

**S1 Table. Social Trust Items**

Question stem: *"How much do you trust each of the following?"*

(Response options: 1 = Do not trust at all, 2 = Do not trust very much, 3 = Neutral, 4 = Trust somewhat, 5 = Trust very much)

| **Item** | **Content** |
| --- | --- |
| 1 | Trust in family |
| 2 | Trust in friends or neighbors (including school seniors/juniors, alumni, classmates, and club members) |
| 3 | Trust in coworkers (including colleagues, supervisors, and subordinates) |
| 4 | Trust in strangers |
| 5 | Trust in the central government |
| 6 | Trust in local government |
| 7 | Trust in corporations |
| 8 | Trust in NGOs and civic organizations |
| 9 | Trust in the media |
