# Supplementary material for: Social trust and depression among young adults in Seoul, South Korea: The moderating effect of traditional gender role attitudes
Source: PLoS One. 2026 Aug 28;21(8):e0355298. doi: 10.1371/journal.pone.0355298 (PMC13524275; doi:10.1371/journal.pone.0355298)
Supplement: Supplementary file 2 [file pone.0355298.s002.docx]

**S2 Table. Traditional Gender Role Attitude Items**

Question stem:

*"Please indicate the response option that best reflects your opinion for each of the following statements."*

(Response options: 1 = Strongly disagree, 2 = Disagree, 3 = Neutral, 4 = Agree, 5 = Strongly agree)

| **Item** | **Content** |
| --- | --- |
| 1 | The primary responsibility for family livelihood lies with men. |
| 2 | Men should avoid showing weakness whenever possible. |
| 3 | Even when employed, the primary responsibility for childcare belongs to women. |
| 4 | Women are better than men at housework, cooking, and caring for others. |
| 5 | Important social affairs should primarily be led by men. |
| 6 | Men should serve as leaders in society. |
