# Supplementary material for: Social trust and depression among young adults in Seoul, South Korea: The moderating effect of traditional gender role attitudes
Source: PLoS One. 2026 Aug 28;21(8):e0355298. doi: 10.1371/journal.pone.0355298 (PMC13524275; doi:10.1371/journal.pone.0355298)
Supplement: Supplementary file 3 [file pone.0355298.s003.docx]

**S3 Table. Gender Differences in Key Study Variables**

| **Variables** | **Women (n=315)** | **Men (n=277)** | **t** | **p** |
| --- | --- | --- | --- | --- |
|  | **Mean±SD** | **Mean±SD** |  |  |
| **Social trust** | 2.77±0.56 | 2.89±0.62 | -2.37 | .018 |
| **Traditional gender role attitudes** | 2.09±0.87 | 2.60±0.91 | -6.90 | <.001 |
| **Depression** | 7.25±6.25 | 5.56±6.07 | 3.32 | **t** |
